# White matter alterations to cingulum and fornix following very preterm birth and their relationship with cognitive functions

**DOI:** 10.1016/j.neuroimage.2017.02.026

**Published:** 2017-04-15

**Authors:** Chiara Caldinelli, Sean Froudist-Walsh, Vyacheslav Karolis, Chieh-En Tseng, Matthew P. Allin, Muriel Walshe, Marion Cuddy, Robin M. Murray, Chiara Nosarti

**Affiliations:** a Department of Psychosis Studies, Institute of Psychiatry, Psychology and Neuroscience, King's Health Partners, King's College London, UK; b Centre for the Developing Brain, Division of Imaging Sciences and Biomedical Engineering, King's Health Partners, King's College London, St. Thomas' Hospital, London, UK

**Keywords:** Preterm birth, Diffusion MRI, Tractography, Memory

## Abstract

Very preterm birth (VPT; <32 weeks of gestation) has been associated with impairments in memory abilities and functional neuroanatomical brain alterations in medial temporal and fronto-parietal areas. Here we investigated the relationship between structural connectivity in memory-related tracts and various aspects of memory in VPT adults (mean age 19) who sustained differing degrees of perinatal brain injury (PBI), as assessed by neonatal cerebral ultrasound. We showed that the neurodevelopmental consequences of VPT birth persist into young adulthood and are associated with neonatal cranial ultrasound classification. At a cognitive level, VPT young adults showed impairments specific to effective organization of verbal information and visuospatial memory, whereas at an anatomical level they displayed reduced volume of memory-related tracts, the cingulum and the fornix, with greater alterations in those individuals who experienced high-grade PBI. When investigating the association between these tracts and memory scores, perseveration errors were associated with the volume of the fornix and dorsal cingulum (connecting medial frontal and parietal lobes). Visuospatial memory scores were associated with the volume of the ventral cingulum (connecting medial parietal and temporal lobes). These results suggest that structural connectivity alterations could underlie memory difficulties in preterm born individuals.

## Highlights

• Very preterm born adults exhibit memory and learning impairments.
• White matter tracts implicated in memory are altered following perinatal brain injury.
• Structural alterations to memory tracts may underlie specific memory impairments.

## Introduction

Very preterm birth (VPT; <32 weeks of gestation) has been associated with neurological, social and emotional problems (Johnson and Marlow, 2014, Van Hus et al., 2014), as well as impairments in several cognitive abilities, including executive function (Burnett et al., 2013, Kroll et al., in press), language processing (Lewis et al., 2000) and various aspects of memory (Nosarti and Froudist‐Walsh, 2016, De Haan, 2010).

Memory is an abstract construct, which refers to the retention of learning or experience (Blakemore, 1988) and is a core component of cognitive function. Key processes involved in memory include (a) registration (or reception), which is closely associated with selective attention, (b) storage, and (c) retrieval. Therefore, memory is one of the elementary processes that is likely to contribute to the development of global cognitive abilities, with a key role in learning (Rose et al., 2004).

Memory deficits have been reported following very preterm birth, as early as at term equivalent (Therien et al., 2004), with childhood studies focusing on working memory and episodic memory (see Nosarti and Froudist‐Walsh, 2016; Anderson, 2014 for recent reviews). Working memory refers to the capacity to temporarily store information for everyday activities, whilst episodic memory refers to remembering specific past events embedded in a spatial and/or temporal framework. Overall, existing evidence suggests that VPT individuals show impairments in episodic memory (Narberhaus et al., 2008, Isaacs et al., 2000) and working memory in childhood and adolescence (Nosarti and Froudist‐Walsh, 2016, Schneider et al., 2014, Thompson et al., 2014). Some studies have additionally reported deficits in visual reproduction tests (Aanes et al., 2015, Nosarti et al., 2014), which require a rapid processing of information and its effective organization for subsequent recall.

From a cognitive neuroscience perspective, different neural networks subserve different memory components. For example, working memory involves predominantly frontal and parietal cortices (D'Esposito, 2007), which are reciprocally connected by the dorsal cingulum (Catani et al., 2013) and superior longitudinal fasciculi (Petrides and Pandya, 2002). On the other hand, the hippocampus is a critical region for episodic memory. The hippocampus lies at the end of convergent processing streams of visual, auditory (from temporal cortex) and spatial information (from parietal areas), and is thus ideally located to bind different types of information in order to form memories of discrete episodes (Rolls, 2015). Other areas that form the episodic memory system include the medial temporal, posterior cingulate and prefrontal, cortices (connected via the cingulum bundle), and the thalamus and mammillary bodies (connected to the hippocampus via the fornix) (Rugg and Vilberg, 2013).

Memory impairments in VPT individuals could be understood in the context of the observed neurodevelopmental structural and functional brain alterations in networks supporting memory functions. For instance, the periventricular location of the hippocampus and its protracted neurogenesis (Sullivan et al., 2001), makes it particularly vulnerable to perinatal brain injury (PBI) associated with very preterm birth, such as intraventricular haemorrhage (IVH; Khwaja and Volpe, 2008).

Reductions in volume of the hippocampus, as well as the interconnected thalamus and posterior cingulate cortex, have been observed at term-equivalent age in VPT infants and have been related to the degree of prematurity (Ball et al., 2012). Smaller bilateral hippocampal volume in VPT samples compared to controls have been described from childhood to adult life (Aanes et al., 2015, Brunnemann et al., 2013, Nosarti et al., 2002), although in some studies significant between-group differences disappeared when correcting for intracranial volume (Omizzolo et al., 2013, Fraello et al., 2011). Hippocampal volume at term has been associated with working memory at age two (Woodward et al., 2005) and with episodic memory abilities at age seven (Thompson et al., 2013, Thompson et al., 2014, Omizzolo et al., 2013). However, in the same study cohort, both hippocampal volume at 7 years of age and longitudinal volumetric changes between term and age 7 were not associated with episodic memory, suggesting that children with early hippocampal damage may have had limited resources for the development of subsequent memory functions.

Structural and functional alterations in the fornix, corpus callosum, superior longitudinal fasciculus, dorsal cingulum and the parahippocampal, entorhinal and perirhinal cortices following VPT birth have been further documented in adolescence and early adulthood and have often correlated with memory ability (Froudist-Walsh et al., 2015, Nosarti et al., 2014, Salvan et al., 2014, Skranes et al., 2012, Allin et al., 2011, Narberhaus et al., 2008).

Here we used diffusion magnetic resonance imaging (MRI) tractography to study white matter fasciculi that are important for memory function: the cingulum, which connects the medio-temporal lobe (enthorinal cortex) to the prefrontal cortex (cingulate gyrus), subdivided into dorsal cingulum (DC; connecting medial frontal and parietal cortex, including the cingulate gyrus) and ventral cingulum (VC; connecting medial temporal and parietal cortex, including the retrosplenial cortex), and the fornix, which is the principal efferent neural pathway of the hippocampus and connects it to the mamillary bodies and the septal region.

We hypothesized that each of these white matter fasciculi would be reduced in volume in VPT young adults, and particularly those who experienced high-grade PBI. This was based on our previous finding that the most severe pervasive and tract-specific white matter alterations following VPT birth were seen in individuals who suffered PBI in the form of intraventricular haemorrhage and ventricular dilatation (Froudist-Walsh et al., 2015, Nosarti et al., 2014). Moreover, we hypothesized that cingulum and fornix alterations would be associated with scores on verbal and visual memory tests which we previously reported as being lower in VPT adults compared to controls (Nosarti et al., 2014, Allin et al., 2011).

## Methods

### Participants

VPT participants were drawn from a cohort of infants who were born before 33 gestational weeks in 1982–1984. All individuals were admitted within 5 days of birth to the Neonatal Unit at University College London Hospital, where they received neonatal cranial ultrasound daily for the first 4 days of life, at 1 week, and weekly until they were discharged from hospital. All infants who survived were enrolled into a longitudinal follow-up study (n=302). Results of other neuroimaging studies in the subject sample which forms the basis of the present study are reported in Nosarti et al. (2014), Nam et al. (2015), and Allin et al. (2011).

At 19–20 years, 94 individuals underwent neuropsychological assessment and 87 received diffusion MRI (Allin et al. 2011). Exclusion criteria were severe visual, hearing and motor impairment. Socio-demographic and neonatal characteristics of the study sample are shown in Table 1. VPT participants were further subdivided on the basis of their neonatal cranial ultrasound classification. Hemorrhages confined to the germinal matrix, and those spreading to the brain parenchyma or lateral ventricles were combined together as periventricular hemorrhage (PVH) (Stewart et al., 1983) and their grade was defined according to Papile et al. (1978). Ventricular dilatation was described as a visible dilatation of one or both lateral ventricles with cerebrospinal fluid, although insufficient to meet the criteria for hydrocephalus. Those VPT participants with normal neonatal cranial ultrasound results (n=30) and with uncomplicated PVH (grade I-II), without ventricular dilatation (n=34) were grouped together and were labelled VPT-N (in total, n=64); those individuals with high-grade PVH (grade III-IV) and/or ventricular dilatation were labelled VPT-PBI (n=20). There were no instances of periventricular leukomalacia. Please refer to Nosarti et al., 2011 for further details on neonatal cranial ultrasound classification. 48 age-matched term-born individuals were also assessed. The inclusion criterion for this group was full term birth (37–42 completed weeks of gestation); exclusion criteria were birth complications (for example, low birth weight <2500, endotracheal mechanical ventilation, prolonged gestation (>42 weeks), severe visual, hearing and motor impairment).

All participants were native English speakers. All participants gave informed written consent, were reimbursed for travel expenses and received a nominal remuneration for participation in the study. The study was given ethical approval by the South London and Maudsley Research and Ethics Committee and by the Psychiatry, Nursing and Midwifery Research Ethics Subcommittee, King's College London.

### Data acquisition

Diffusion-weighted imaging was acquired on a 1.5 T GE Signa MR scanner (GE Healthcare, USA) at the Institute of Psychiatry/Maudsley Hospital, King's College London. A total of 60 contiguous near-axial slices were acquired with no gap and the following parameters: isotropic voxels of 2.5 mm×2.5 mm×2.5 mm, reconstructed as 1.875 mm×1.875 mm×2.5 mm, with coverage of the whole head, peripherally-gated to the cardiac cycle, with an echo time of 107 ms, and effective repetition time of 15 R-R intervals; diffusion encoding gradients duration 17.3 ms, providing a maximum diffusion weighting of 1300 s/mm^2^ (Jones et al., 2002). At each slice location, 7 images with no diffusion gradient (i.e., b = 0 s/mm^2^) were acquired, together with 64 diffusion weighted images (1300 s/mm^2^) with gradient directions distributed evenly in space.

T1-weighted MR images were also collected. A total of 124 slices were acquired, with a matrix size of 256×256, slice thickness of 1.5 mm and slice gap of 0 mm. Images were acquired in coronal plane, with a spoiled gradient recalled pulse sequence (flip angle 35°, field of view 240 mm, echo time 5 ms, repetition time 35 ms).

### Data processing

Diffusion-weighted images were processed according to the methods described in Froudist-Walsh et al. (2015). In particular, brain masks were created manually using the Brain Extraction Tool (BET) of FMRIB Software Library (FSL), in order to delete non-brain tissue from an image of the whole head (Smith, 2002). Motion and eddy-current correction was performed on the masked diffusion data using ExploreDTI (Leemans et al., 2009).

A spherical deconvolution approach was chosen to allow for the estimation of multiple fibre directions within a single voxel. Whole brain tractography was performed using a damped version of the Richardson-Lucy algorithm (Dell'Acqua et al., 2010). Parameters were optimised in order to find the best possible balance between resolving multiple fibre directions and creating minimal spurious (false-positive) fibre orientation distribution (FOD) components. After carefully altering the parameters and testing on multiple subjects, a regularisation threshold of *η*=.2 was used; a fibre response function=2; 300 algorithm iterations and regularisation parameter v=20. Fibre orientation estimates were taken from the orientation of the peaks of the FOD profile. We applied an “absolute” (equal to four times the amplitude of a spherical FOD obtained from a grey matter voxel) and a “relative” threshold (equal to 8% of the amplitude of the maximum amplitude of the FOD at that voxel) at each voxel to remove the general noise floor and surviving noise local maxima, respectively.

### Tractography algorithm and dissections

Whole-brain tractography was performed in native diffusion space using each FOD peak (that survived thresholding) as a seed. Fibre orientation streamlines were propagated using Euler integration with a step-size of 1 mm. Propagation stopped if the track reached an FOD peak that did not survive thresholding, or if the angle between two successive steps exceeded 60°. Spherical deconvolution, fibre-orientation estimation and tractography were performed using StarTrack (Dell'Acqua et al., 2010).

**Figure 1:**
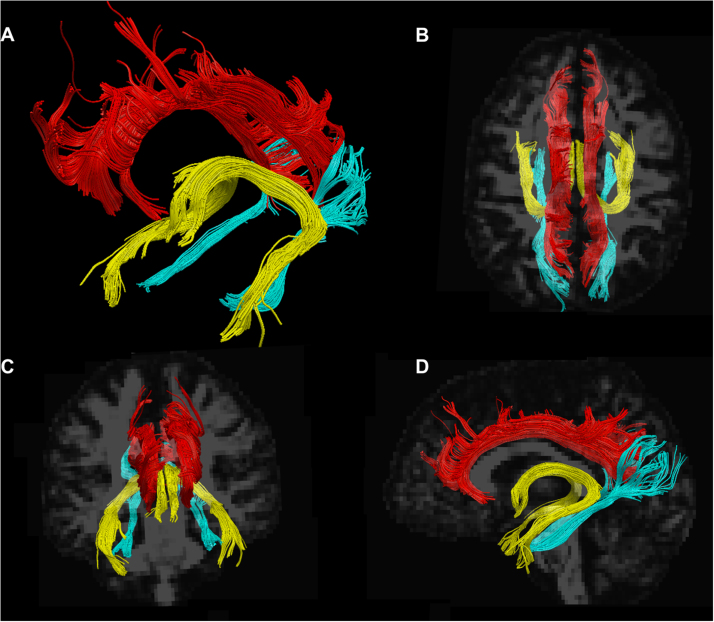
A is an example of dissected tracts in a study participant: dorsal cingulum is displayed in red, ventral cingulum in sky blue and fornix in yellow. B, C and D show dorsal, frontal and lateral views, respectively. (For interpretation of the references to color in this figure legend, the reader is referred to the web version of this article.)

Virtual dissections were manually performed in TrackVis (trackvis.org) using a two-region method to isolate single tracts (Catani and Thiebaut de Schotten, 2008, Froudist-Walsh et al., 2015). The cingulum was divided into dorsal and ventral segments, connecting the medial parietal region with the medial frontal and medial temporal lobe, respectively. Only the fibres connecting the medial temporal lobe and parietal lobe (primarily precuneus and retrosplenial cortex) were included in the ventral cingulum. The fornix was dissected in each hemisphere separately and its commissure was not included (Figure 1). All tracts presented were dissected in both hemispheres and the data are presented as the average of the two hemispheres, as there is no strong hypothesis of a lateralization of memory abilities following preterm birth.

Artefactual fibres were carefully removed through the use of a manually drawn exclusion region.

In one participant from the VPT-PBI group the right fornix was not recognisable during in vivo dissection and therefore only the left fornix, instead of the average between the two hemispheres, was included in the analysis.

The measures resulting from this processing stream are i) volume and ii) mean hindrance modulated orientational anisotropy (HMOA) of each dissected tract. HMOA represents the absolute amplitude of each lobe of the fiber orientation distribution normalized to a reference amplitude (the highest possible diffusion value in a biological tissue) and is thought to reflect the microstructural properties specific to single fiber populations, even in voxels that contain crossing fibers. HMOA has a range of 0–1, with 1 as the maximum diffusivity, and 0 as an absence of a fiber (Dell'Acqua et al., 2013). Furthermore, mean fractional anisotropy (FA) and mean Radial Diffusivity (RD) maps were obtained with ExploreDTI (Leemans et al., 2009), for each dissected tract.

Intracranial volume (ICV) was calculated using the Voxel Based Morphometry (VBM8) toolbox (http://dbm.neuro.uni-jena.de/vbm/) in Statistical Parametric Mapping (SPM8; http://www.fil.ion.ucl.ac.uk/spm/software/spm8/), as described in Nosarti et al. (2014). Specifically, images were segmented into grey matter, white matter and cerebrospinal fluid using a modified Gaussian Mixture Model to match each voxel to prior probability maps (Ashburner and Friston, 2003). Grey matter, white matter and cerebrospinal fluid total ml units were summed to obtain total ICV. Critically, segmentation into grey matter, white matter and cerebrospinal fluid may have been problematic for participants with marked ventricular dilatation.

### Neuropsychological testing

#### Verbal learning

The California Verbal Learning Test (CVLT; Delis et al., 1987) is a standardized and validated list-learning test. It assesses the quantity of information recalled and recognized, the strategies used in learning and remembering and the types of recall and recognition errors made. The following summary measures were used:

*List A Short Delay Free Recall*, which measures short term memory and will be referred to as ‘Short-term memory’.

*List A Long Delay Free Recall*, which measures long term memory and will be referred to as ‘Long-term memory’.

*Perseverations*, a measure of repetitions of previously given responses within the same trial. Shortly intervalled errors are called proximal perseverations - often observed in frontal-system pathology. On the other hand, distal perseveration errors refer to repeated errors with a considerable interval between them, a pattern often observed in patients with amnesic or attention deficits (Delis et al., 1987).

*List B*, which measures how many words from a second list of words participants can recall, allowing measurement of the effects of prior learning (list A) on subsequently learned materials (list B), defined as proactive interference (Numan et al., 2000).

*Comparison of Short Delay Cued Recall and Short Delay Free Recall.* If scores on the cued recall condition are worse than scores on the free recall condition, the examinee may not have been proficient at using a semantic cluster comparison between free recall and category-cued recall. If the examinee scores worse on the free recall condition than the cued recall condition, then deficits in retrieval may be present (this pattern is often found in individuals with recently diagnosed Huntington's disease (Delis et al., 1987)). This score will be referred to as ‘Short term memory strategy’.

*Difference between Long Delay Cued Recall and Long Delay Free Recall.* An assessment of the type of learning strategy used for items in long term memory. This score will be referred to as ‘Long term memory strategy’.

#### Visuospatial memory

The Visual Reproduction test of the Wechsler Memory Scale-Revised (WMS-R) (Wechsler, 1987) was used to assess visuospatial memory. Participants were asked to reproduce non-verbal stimuli, either just after these were shown (immediate picture recall; WMS-R Copy) or after a delay (delayed picture recall; WMS-R Delay).

#### General intellectual functioning

The Wechsler Abbreviated Scale of Intelligence (WASI; Wechsler, 1999) was used to provide estimates of age-standardized verbal (vocabulary and similarities subtests), performance (block design and matrix reasoning subtests) and full-scale IQ (calculated as a mean score of verbal and performance IQ).

### Statistical analysis

Analyses were conducted using IBM SPSS (IBM Corp. Released 2013. IBM SPSS Statistics, Version 22.0. Armonk, NY: IBM Corp). Tract volumes were corrected for intracranial volume by using robust regression and a logistic weight function in Matlab 7.8 (http://www.mathworks.co.uk/products/matlab/). Gaussian distribution of the data, which was assessed using the Shapiro-Wilk test (Shapiro and Wilk, 1965), was not confirmed for all variables in our samples. Therefore, Kruskal-Wallis (Kruskal and Wallis, 1952) tests were used to perform analysis of variance. Post hoc analyses were performed with Dunn-Bonferroni tests (Dunn, 1959). Two-tailed partial Spearman rank correlations (Spearman, 1904) were performed between the measurements of each dissected single tract (corrected for ICV) and each individual memory score. The correlations were performed across all groups and, additionally, within single groups, with the caveat that the small within-group sizes may not be sufficient to detect the brain-behaviour associations of interest, due to a small range of scores.

Results of Kruskal-Wallis tests and Spearman rank correlations were corrected for false discovery rate, using the Benjamini and Hochberg correction (FDR; p<0.05) (Benjamini and Hochberg, 1995), separately for each assessed domain. The adjusted rank transform test (ART) (Leys and Schumann, 2010) was used to test for interactions between tracts, using R statistical package (R Core Team, 2013). The effect sizes were calculated as Cohen's *d* using means and standard deviations [Cohen, 1988].

## Results

### Sample characteristics

Table 1 shows participants' neonatal and socio-demographic characteristics as well as IQ. The VPT-PBI group had younger gestational age and lower birth weight compared to the VPT-N group. The VPT-N group was slightly older than the control group. The VPT-N group had lower full scale IQ than controls. The three groups did not differ in parental socio-economic status.

Educational data were available for 116 participants (90%). Every participant in the control group (N=41; 100%) was in full-time education either at university or in the process of obtaining their A-levels. The majority of VPT participants (N=70; 93%) were in full-time education, whereas a minority (N=5; 6.7%) were receiving vocational training. In the UK, students are required to complete A-level exams, the equivalent of matriculation exams, prior to attending university. Some students may choose to complete a National Vocational Qualification (NVQ) which are work based awards instead.

### White matter tracts analysis

**Figure 2:**
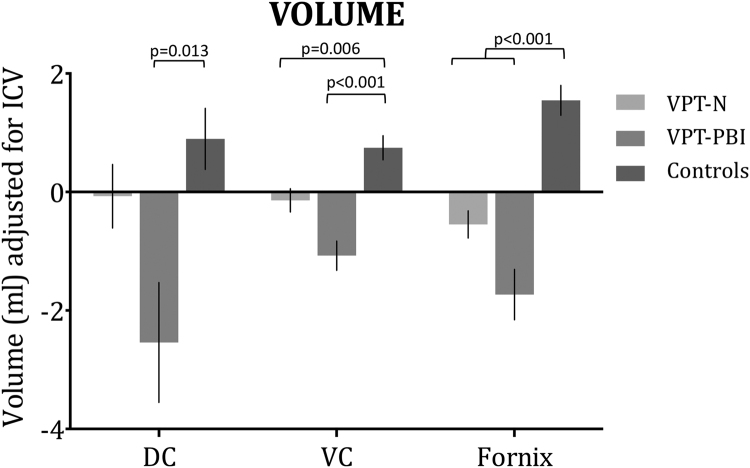
Mean and standard error of the mean of single tract volumes (ml, adjusted for ICV), by group.

ICV was unavailable for 2 participants in the VPT-N group and 1 control, due to poor quality of T1-weighted MR images; these participants were excluded from subsequent analysis. One-way analysis of variance by ranks (Kruskal-Wallis test) revealed significant volumetric group differences in all three tracts of interest: DC, VC and fornix (see Table 2). Post-hoc analyses revealed that the VPT-PBI group had significantly smaller volume of all three tracts compared to controls (DC: H=−28.43 p=0.013; VC: H=−44.103, p<0.001; fornix: H=−56.59, p<0.001). The VPT-N group had significantly smaller volume of VC and fornix compared to controls (H=−22.295, p=0.006 and H=−37.17, p<0.001, respectively). These results are summarised in Figure 2.

There was a significant group*tract interaction (T(4)=3.886, p=0.001, surviving FDR correction). To further investigate the greater difference between the groups in some tracts compared to others, post hoc tests were performed. Results revealed that the difference in tract volumes between VPT-PBI and controls and VPT-N and controls was significantly different between VC and fornix (respectively T(1)=96.654, p<0.05 and T(1)=72.130, p<0.05).

**Figure 3:**
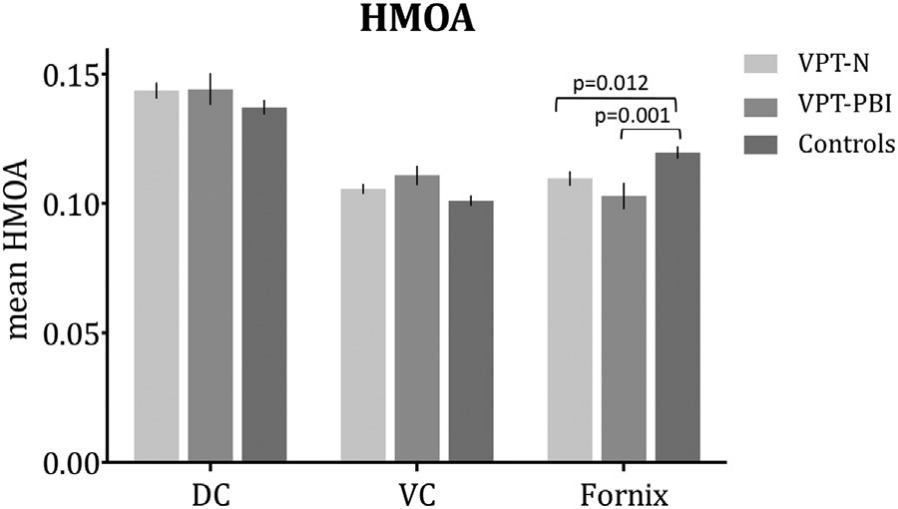
Mean of single tract hindrance modulated orientational anisotropy (HMOA), by group. HMOA range is 0–1, with 1 as the maximum diffusivity, and 0 as an absence of a fiber.

One-way analysis of variance by ranks (Kruskal-Wallis test) revealed significant between group differences in fornix FA, which did not survive FDR correction. No significant between group differences were found in mean RD. Significant between group differences were however evident in fornix HMOA (surviving FDR correction). Post hoc analysis revealed that the VPT-PBI had smaller fornix HMOA than controls (H=−37.045, p=0.001) and VPT-N had significantly smaller fornix HMOA than controls (H=−20.809, p=0.012 Figure 3). Results are summarised in Table 3.

Mean HMOA correlated positively with mean FA of all the three tracts (DC: r=0.86, VC: r=0.76, fornix: r=0.89, all p<0.0001) and negatively with mean RD of all the three tracts (DC: r=−0.65, p<0.0001; VC: r=−0.52, p<0.0001; fornix: r=−0.23, p=0.008), across all groups. All the above results survived FDR correction.

Mean HMOA correlated positively with fornix volume (fornix: r= 0.52, p<0.0001, surviving FDR correction) and did not significantly correlate with DC or VC volume (DC: r=−0.15 p=0.083; VC: r=−0.06 p=0.524).

Effect size calculation showed that in the VPT-PBI group the alterations in fornix HMOA, volume of fornix, DC and VC had effect sizes of −0.6, −0.8, −1.5 and −1.8 respectively, indicating medium to very large effects; while in the VPT-N group the volume of the fornix had an effect size of −1.2, indicating a very large effect, fornix HMOA and VC volume both had an effect size of −0.6, indicating a medium effect.

### Neuropsychological performance

**Figure 4:**
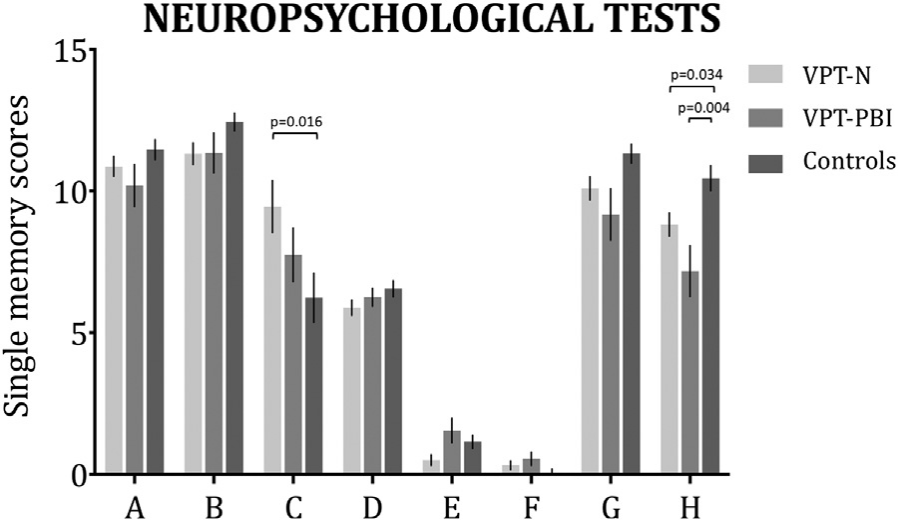
Mean and standard error of the mean of selected CVLT and WMS-R scores, by group. A=Short-term memory B=Long-term memory; C=Perseverations; D=List b; E=Short-term memory strategy; F=Long-term memory strategy; G=WMS-R Copy; H=WMS-R Delay.

When considering specific CVLT summary scores (described in Methods), significant between group differences were observed in Perseverations and Short-term memory strategy. Significant between-group differences were also found in WMS-R Delay and full-scale IQ. Results are shown in Table 4 and Figure 4. Post hoc analyses showed that the control group performed better than the VPT-N group in CVLT Perseverations (H=20.024, p=0.016), WMS-R Delay (H=−17.753, p=0.034) and full scale IQ (H=−24.489, p=0.002). The control group also had higher scores than the VPT-PBI group in WMS-R Delay scores (H=−32.761, p=0.004).

### Tract volumes and neuropsychological performance

**Figure 5:**
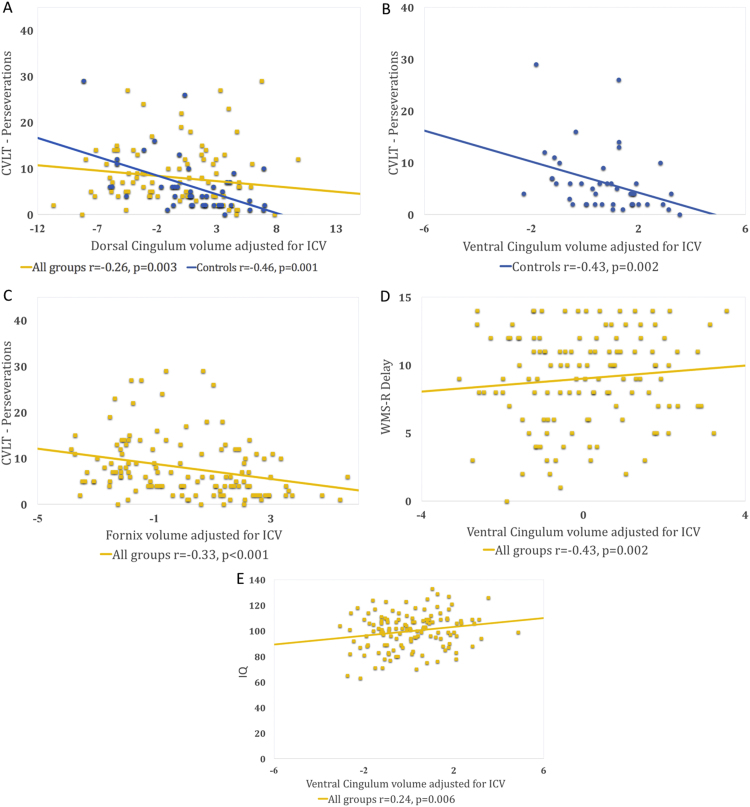
Significant correlations between single tract volumes (adjusted for ICV) and cognitive measures after FDR correction.

As CVLT Perseverations scores, WMS-R Delay scores and full-scale IQ differed significantly between the groups, we investigated the relationship between these measures and single tract volume (corrected for ICV) FA, RD and HMOA across all sample groups. Statistical tests that survived FDR correction included a correlation between higher CVLT Perseverations scores (i.e. more errors) and smaller DC and fornix volumes, and higher WMS-R Delay scores (i.e. more correct responses) and larger VC volume. Higher IQ scores significantly correlated with larger VC volume (see Table 5 and Figure 5).

Correlation analyses between tract-specific FA, RD and HMOA and neuropsychological measures did not reveal any statistically significant results.

To further investigate the relationship between tract volumes and neuropsychological scores, we performed within group correlation analyses. In the VPT-N group CVLT Perseverations correlated with fornix volume (r=−0.278, p=0.028, not surviving FDR correction). In the control group CVLT Perseverations significantly correlated with both DC and VC volume (respectively r=−0.456 p=0.001 and r=−0.431 p=0.002, both surviving FDR correction) and IQ with VC volume (r=0.315, p=0.031, not surviving FDR correction). See Table 6 and Figure 5.

## Discussion

The results of our study are in line with previous findings that showed a significant association between very preterm birth and alterations in brain structural connectivity, which have important consequences for neurocognitive development (Karolis et al., 2015; Fischi-Gomez et al., 2015; Salvan et al., 2014; Ball et al., 2012). Firstly, our results showed that the fornix, DC and VC were reduced in volume in VPT young adults, particularly in those who experienced severe perinatal brain injury, as assessed by neonatal cranial ultrasound. Secondly, they suggested that performance on specific aspects of memory, i.e., those involving the encoding of information during learning, was worse in VPT young adults than controls, and lower memory scores were selectively associated with alterations in DC, VC and fornix. These findings echo those of our recent functional MRI studies which suggested altered brain activation patterns during learning and memory tasks in adults born very preterm (Froudist-Walsh et al., 2015, Brittain et al., 2014, Salvan et al., 2014), which were associated with structural alterations to white matter tracts including the fornix (Salvan et al., 2014, Tseng et al., 2017) and dorsal cingulum (Froudist-Walsh et al., 2015, Tseng et al., 2017).

Very preterm born adults who had sustained PBI had smaller cingulum volume (both VC and DC) than controls. This suggests that early periventricular brain injury may result in a certain amount of cell death in areas projecting through the cingulum bundle, leading to a permanent reduction in the volume of these tracts. However, in the VPT-N group (those with normal or less severe neonatal ultrasound classification), only VC volume was significantly smaller compared to controls. Such findings indicate that the ventral cingulum may be vulnerable to even minor brain insults associated with very preterm birth, whereas the dorsal cingulum may be only affected in cases of high grade PVH (VPT-PBI group), perhaps due to this structure being separated from the ventricles by the corpus callosum. Our current analyses did not detect any significant between-group differences in terms of cingulum microstructure. We speculate that, in spite of volumetric alterations associated with PBI, it is possible that the surviving fibre bundles go on to develop typical microstructural attributes, resulting in relatively normal HMOA, FA and RD measures. Additionally, the lack of correlation between HMOA and volume of both sections the cingulum could be explained by the fact that at the relatively low b-values used in this study, HMOA is likely to reflect a combination of macro and microstructural white matter features. In a thinner tract like the fornix, it is possible that HMOA is more closely related to the macrostructural features, like volume fraction, than in the DC and VC (Dell'Acqua et al., 2013, Raffelt et al., 2012).

On the other hand, individuals born VPT, independent of their type of neonatal ultrasound classification, had smaller fornix volume and lower HMOA compared with controls. Periventricular regions, such as the fornix, that are likely to be affected by hypoxia/ischemia (Stone et al., 2008), often show volumetric and functional alterations in VPT-born samples compared to controls. FA and RD were not significantly different between groups for any of the dissected tracts, which leads us speculate that HMOA may be more sensitive to microstructural white matter changes, as has previously been suggested by simulations (Dell'Acqua et al., 2013). These findings in the fornix could reflect the long term-consequence of early damage to glial precursor cells, which are not fully developed by the time very preterm infants are born (e.g., before the third trimester of gestation), which could cause loss of oligodendroglia (Khwaja and Volpe, 2008).

At a cognitive level, the results of this study show that our VPT participants did not have a pervasive deficit in short or long term verbal memory, even if they had sustained severe perinatal brain injury. However, they scored lower than controls on measures of efficient short-term learning and organizing strategies (WMS-R Delay) and had the tendency to repeat the same mistakes (CVLT Perseverations), a function that mainly relies on working memory (Stedron, Sahni, and Munakata, 2005). Such results may suggest a disturbance in the way the learning occurs, affecting the encoding of information, thus causing subsequent difficulties in retrieving stored material.

Additionally, IQ was lower in the VPT-N group compared to controls. The positive correlation between IQ and VC and fornix volume suggests that these tracts are important for general intellectual functioning, in line with the hypothesis that specific aspects of memory (e.g., working memory indirectly assessed here via CVLT Perseverations) are closely related to IQ (Ackerman, Beier and Boyle, 2005).

The current results are in line with the observations of a previous functional MRI study from our group, which investigated the dynamic formation of visual memory associations and demonstrated reduced recruitment of the hippocampus, parahippocampal and posterior cingulate cortices in VPT adults compared to controls during encoding (Brittain et al., 2014). Altered engagement of the neural substrates of learning might explain the lower scores that VPT participants showed on the WMS-R Delay task, leading to less effective organization of information for subsequent recall. Furthermore, impairments in specific aspects of learning in VPT individuals seen here may result from deficits in other (unmeasured) cognitive functions that have been described in VPT children, including visuospatial abilities and selective aspects of attention (Marlow et al., 2007, Mulder et al., 2009).

Participants' scores on measures reflecting less effective learning strategies correlated with smaller volumes of the chosen white matter fasciculi. Specifically, smaller DC volume was associated with more persisting errors (CVLT Perseverations), while smaller VC volume was associated with lower full-scale IQ. Such results suggest that the two subdivisions of the cingulum (dorsal and ventral) may underpin different aspects of mnemonic processing. Results of animal studies have shown differences between DC and VC in their axonal population (Mufson and Pandya, 1984), while research in healthy volunteers demonstrated significant differences in subregional white matter properties including radial diffusivity and fractional anisotropy (Jones et al., 2013). These findings support the hypothesis that the DC and VC contain connections which are critical for various functions, and that the white matter properties of each subdivision are optimized in order to perform specific operations. On a methodological note, Jones et al. (2013) proposed an alternative subdivision of the cingulum to the one used in this study. Instead of two subdivisions they suggested three: a parahippocampal, a retrosplenial and a subgenual section; and provided evidence that, divided in this manner, the tract sections differed in medial/lateral location as well as some DTI-based metrics. It remains to be seen which scheme for subdividing the cingulum provides greater predictive value for neuropsychological performance. Indeed, in a more recent study, the same group appears to have chosen a subdivision of the cingulum very similar to the one used here (Bracht, et al., 2015). Future studies should look at the lateralisation of these tracts, and how that may relate to the recruitment of grey matter structures during cognitive tasks.

Our results of significant associations between DC volume and test scores reflecting efficient learning strategies highlight the crucial role of white matter tracts connecting the frontal cortex and parietal areas in supporting the formation of specific learning strategies (Hirst and Volpe, 1988), including strategic retrieval (Ullman et al., 2014) and cognitive control (Catani et al., 2013). On the other hand, significant associations between the VC and visuospatial processing may reflect the role of this white matter tract in connecting parahippocampal regions to restrosplenial cortex and medial parietal brain areas involved in spatial orientation and memory (Rolls, 2015).

The presence of persisting errors (CVLT Perseverations) was also associated with smaller fornix volume, reflecting the role of the hippocampal network in learning and memory. In a similar cohort of VPT young adults to that studied here we previously reported that white matter volume in a large cluster comprising sections of posterior corpus callosum, thalamus and fornix correlated with WMS-R Delay scores (Nosarti et al., 2014) and that activation in these areas during a learning task was associated with altered fornix microstructure (Salvan et al., 2014).

To conclude, the present study indicates that the effects of VPT birth on white matter anatomy and memory functioning last until young adulthood and are associated with the extent of perinatal brain injury. VPT individuals who suffered PBI, in particular, showed reductions in volume of white matter tracts that were associated with scores on tests assessing visuospatial memory and efficient organization of information for subsequent recall, as well as the tendency to repeat mistakes. These results suggest that structural alterations to memory-related tracts may underpin the lower scores on specific aspects of memory observed in VPT young adults. Understanding the neural correlates of the specific memory and learning mechanisms in VPT individuals, as well as being able to identify early predictors of outcome, may aid the development of preventative strategies aimed at attenuating the neurodevelopmental sequelae of VPT birth.

## Figures and Tables

**Table 1:** Participants' neonatal, socio-demographic characteristics and IQ, by group.

	**VPT-N**	**VPT-PBI**	**Controls**	**Statistics**
	**(n=64)**	**(n=20)**	**(n=48)**	
**Gestational age, weeks**^a^	29.20	27.65	40.10	H=7.482
				p=0.006
**Birth weight, grams**^a^	1328.22	1060.25	3310.54	H=7.775
				p=0.005
**Males/Females**	35/29	10/10	26/22	X^2^=2.423
				p=0.659
**Age at assessment**^b^	19.8 (1.24)	19.4 (0.68)	19.1 (1.24)	H=10.525
				p=0.005
				
**SES**^c^				X^2^=5.292
				p=0.507
**I-II**	28 (43.8%)	9 (45%)	27 (7%)	
**III**	25 (39.1%)	8 (40%)	12 (25%)	
**IV-V**	9 (14.1%)	3 (15%)	9 (18.8%)	
**Unclassified**	2 (3.1%)	0 (0%)	0 (0%)	
**Full scale IQ**^d^	96	98.47	105.81	H=11.828
	(12.71)	(15.98)	(13.85)	p=0.003

a VPT-N compared to VPT-PBI.

b Information was missing for 1 control participant and 2 participants from VPT-N group. Statistically significant differences were found between the VPT-N and control groups (H=21.744, p=0.008).

c Parental socio-economic status (SES) was measured by the Classification of Occupation (CO80, Her Mayesty’ Stationery Office, 1980), with I as the highest and V as the lowest socio-economic group.

d Statistically significant differences were found between the VPT-N and control groups (H=−23.66, p=0.003).

**Table 2:** Mean tract volume (ml) adjusted for ICV, standard deviation and statistics, by group. The white matter tracts shown are from three representative participants (one for each group).

**Table 3:** Mean, standard deviation and statistics of single tract FA, RD and HMOA, by group.

		**VPT-N**	**VPT-PBI**	**Controls**	**Kruskal-Wallis test**
**DC**	**FA**	0.402139	0.397428	0.400746	H=0.642
		(±0031012)	(±0.037708)	(±0.028266)	p=0.725
	**RD**	0.000653	0.0006514	0.0006513	H=0.115
		(±0.000049)	(±0.000036)	(±0,000029)	p=0.944
	**HMOA**	0.143752	00.144245	0.137234	H=1.737
		(±0.022135)	(±0.026135)	(±0.017665)	p=0.420
					
**VC**	**FA**	0.382188	0.392278	0.375755	H=2.365
		(±0,032660)	(±0.043451)	(±0.026349)	p=0.307
	**RD**	0.000723	0.000711	0.000726	H=1.550
		(±0.000039)	(±0.000043)	(±0.000036)	p=0.461
	**HMOA**	0.105668	0.110913	0.101136	H=6.654
		(±0.012822)	(±0.015463)	(±0.012096)	p=0.036*
					
**Fornix**	**FA**	0.385063	0.367289	0.394804	H=7.253
		(±0.040337)	(±0,045410)	(±0.0042)	p=0.027*
	**RD**	0.001844	0.000978	0.000986	H=0.245
		(±0.006761)	(±0.000121)	(±0.000062)	p=0.885
	**HMOA**	0.109706	0.102931	0.119781	**H=15.968**
		(±0.020029)	(±0.021807)	(±0.014934)	**p<0.001**§

* Not significant after FDR correction.

§ Significant after FDR correction.

**Table 4:** Mean, standard deviation and statistics for selected CVLT and WMS-R measures, by group.

	**VPT-N**	**VPT-PBI**	**Controls**	**Kruskal-Wallis test**
**CVLT Short-term memory**	10.87	10.20	11.47	H=1.948
	(±2.77)	(±3.35)	(±2.43)	p=0.38
**CVLT Long-term memory**	11.32	11.35	12.45	H=3.123
	(±3.02)	(±3.15)	(±2.17)	p=0.21
**CVLT Perseverations**	9.45	7.75	6.23	**H=8.082**
	(±7.21)	(±4.25)	(±5.93)	**p=0.018**§
**CVLT List B**	5.89	6.25	6.55	H=3.556
	(±2.1)	(±1.41)	(±1.95)	p=0.169
**CVLT Short-term memory strategy**	0.5	1.55	1.15	H=6.031
	(±1.47)	(±1.93)	(±1.59)	p=0.049
**CVLT Long-term memory strategy**	0.32	0.55	0.06	H=4.154
	(±1.23)	(±1.05)	(±0.85)	p=0.125
**WMS-R Copy**#	10.1	9.18	11.33	H=5.212
	(±3.24)	(±3.75)	(±2.26)	p=0.074
**WMS-R Delay**#	8.82	7.18	10.46	**H=12.15 1**
	(±3.27)	(±3.71)	(±3)	**p=0.002**§

§ Significant after FDR correction.

# WMS-R scores were unavailable for 3 participants belonging to the VPT-PBI group.

**Table 5:** Significant correlations between memory measure that showed a difference between the groups and single tract volume (corrected for ICV), across all sample groups.

	**CVLT Perseverations**	**WMS-R Delay**	**Full-scale IQ**
**DC volume**	**r=−0.256**	r=0.112	r=0.089
	**p=0.003**§	p=0.212	p=0.321
**VC volume**	r=−0.198	**r=0.277**	**r=0.241**
	p=0.024	**p=0.002**§	**p=0.006**§
**Fornix volume**	**r=−0.334**	r=0.177	r=0.170
	**p<0.001**§	p=0.048	p=0.056

§ Significant after FDR correction.

**Table 6:** Significant correlations between memory measure that showed a difference between the groups and single tract volume (corrected for ICV), within group.

	**CVLT Perseverations**	**WMS-R Delay**	**Full-scale IQ**
**VPT-PBI DC volume**	r=−0.222	r=−0.291	r=0.176
	p=0. 347	p=0.257	p=0. 472
**VPT-PBI VC volume**	r=−0.119	r=0.053	r=−0.010
	p=0.616	p=0. 840	p=0.969
**VPT-PBI Fornix volume**	r=−0.053	r=0.065	r=0.204
	p=0.824	p=0. 803	p=0.403
**VPT-N DC volume**	r=−0.163	r=0.102	r=0.103
	p=0. 207	p=0.429	p=0.428
**VPT-N VC volume**	r=0.065	r=0.187	r=0.128
	p=0.616	p=0. 147	p=0.325
**VPT-N Fornix volume**	r=−0.278	r=0.380	r=−0.028
	p=0.028	p=0.766	p=0.828
**Controls DC volume**	**r=−0.456**	r=−0.075	r=−0.078
	p=0.001*	p=0.619	p=0.604
**Controls VC volume**	**r=−0.431**	r=−0.130	r=0.315
	p=0.002*	p=0.388	p=0.031
**Controls Fornix volume**	r=−0.279	r=−0.043	r=0.077
	p=0.058	p=0. 774	p=0.606

* Significant after FDR correction.
